# Supplementary material for: Increased flow rate of hyperpolarized aqueous solution for dynamic nuclear polarization-enhanced magnetic resonance imaging achieved by an open Fabry–Pérot type microwave resonator
Source: Magn Reson (Gott). 2020 Nov 18;1(2):275–84. doi: 10.5194/mr-1-275-2020 (PMC10500708; doi:10.5194/mr-1-275-2020)
Supplement: The supplement related to this article is available online at: https://doi.org/10.5194/mr-1-275-2020-supplement. [file mr-1-275-supplement.zip › mr-1-275-2020-supplement-title-page.pdf]

Supplement of Magn. Reson., 1, 275–284, 2020
https://doi.org/10.5194/mr-1-275-2020-supplement
© Author(s) 2020. This work is distributed under
the Creative Commons Attribution 4.0 License.

Open Access
MAGNETIC RESONANCE

Supplement of

Increased flow rate of hyperpolarized aqueous solution for dynamic nuclear polarization-enhanced magnetic resonance imaging achieved by an open Fabry–Pérot type microwave resonator

Alexey Fedotov et al.

Correspondence to: Thomas F. Prisner (prisner@chemie.uni-frankfurt.de)
and Vasyl Denysenkov (denysenkov@em.uni-frankfurt.de)

- mr-1-275-2020-supplement-title-page.pdf
- Fabry_Perot resonator design.dwg
- Fabry_Perot resonator simulation.cst

The copyright of individual parts of the supplement might differ from the CC BY 4.0 License.
